# Early pregnancy affects expression of Toll-like receptor signaling members in ovine spleen

**DOI:** 10.1590/1984-3143-AR2021-0009

**Published:** 2021-08-06

**Authors:** Leying Zhang, Gengxin Yang, Qiongao Zhang, Pengfei Feng, Meihong Gao, Ling Yang

**Affiliations:** 1 Department of Animal Science, School of Life Sciences and Food Engineering, Hebei University of Engineering, Handan, China

**Keywords:** toll-like receptors, pregnancy, spleen, sheep

## Abstract

Toll-like receptors (TLRs) are involved to the maternal immune tolerance. The spleen is essential for adaptive immune reactions. However, it is unclear that early pregnancy regulates TLR-mediated signalings in the maternal spleen. The purpose of this study was to investigate the effects of early pregnancy on expression of TLR signaling members in the ovine spleen. Ovine spleens were collected at day 16 of the estrous cycle, and at days 13, 16 and 25 of pregnancy (n = 6 for each group). Real-time quantitative PCR, western blot and immunohistochemistry analysis were used to detect TLR signaling members, including TLR2, TLR3, TLR4, TLR5, TLR7, TLR9, myeloid differentiation primary-response protein 88 (MyD88), tumor necrosis factor receptor associated factor 6 (TRAF6) and interleukin-1-receptor-associated kinase 1 (IRAK1). The results showed that expression levels of TLR2, TLR4 and IRAK1 were downregulated, but expression levels of TLR3, TLR5, TLR7, TLR9, TRAF6 and MyD88 were increased during early pregnancy. In addition, MyD88 protein was located in the capsule, trabeculae and splenic cords of the maternal spleen. This paper reports for the first time that early pregnancy has effects on TLR signaling pathways in the ovine spleen, which is beneficial for understanding the maternal immune tolerance during early pregnancy.

## Introduction

The conceptus has half of maternal genetic materials, so conceptus must modify maternal immune system to prevent it from the immune destruction. Interferon-tau (IFNT) is a secretory product of ruminant conceptus, and inhibits prostaglandin F2α (PGF2α) production in the maternal endometria through a paracrine manner to rescue corpus luteum (CL) during early pregnancy (Imakawa et al., 2017). In addition, IFNT also has endocrine actions on extrauterine cells and tissues, including peripheral blood mononuclear cells, which are necessary for enhancing the reproductive efficiency in ruminants (Hansen et al., 2017). During early pregnancy in sheep, IFNT induces upregulation of interferon-stimulated gene (ISG) 15-kDa protein (ISG15) in the CL and liver (Oliveira et al., 2008; Bott et al., 2010; Yang et al., 2020). Furthermore, expression levels of ISGs in the immune tissues, including bone marrow (Yang et al., 2017), thymus (Zhang et al., 2018, 2020b), spleen (Wang et al., 2019; Yang et al., 2018b), and lymph node (Yang et al., 2019; Zhang et al., 2020a), are upregulated during early pregnancy in sheep. Therefore, early pregnancy has effects on the maternal spleen.

As the largest secondary immune organ in the body, the spleen participates in maintenance of specific B cell population that is necessary for initiating immune reactions to blood-borne antigens in mice (Brendolan et al., 2007). The infiltration of immune cell into the uterus induces the sensitized cell migration to the spleen, which leads to the T-cell dependent B-cell responses in the maternal spleen of mice (Hegde et al., 2001). Pregnancy induces changes in the size and expression levels of erythroid-associated genes in the maternal spleen of the rats and mice (Bustamante et al., 2008). Recently, it is reported that the expression levels of progesterone (P4) receptor isoforms, cyclooxygenase-2, PGF synthase, ISGs, tumor necrosis factor (TNF) beta, interleukin (IL)-2, IL-4, IL-5, IL-6, IL-10, gonadotropin releasing hormone and its receptor are upregulated in the ovine spleen (Cao et al., 2021; Li et al., 2019; Wang et al., 2019; Yang et al., 2018a, b) during early pregnancy. In addition, proteins of melatonin receptor 1 and 2 are decreased in the spleen during early pregnancy in sheep (Bai et al., 2020). Therefore, the signaling pathways of immune regulation may change in maternal spleen during early pregnancy.

Toll-like receptors (TLRs) participate in the activation of innate immune system, and antigen-specific adaptive immunity. TLR-mediated signaling pathways include myeloid differentiation primary-response protein 88 (MyD88) dependent pathways and toll/IL-1 receptor (TIR) domain-containing adaptor inducing IFN-β (TRIF) dependent pathway (Kawai and Akira, 2010). TLR ligands induce tolerance through transcriptional regulators of tolerance, and re-programming the innate immune responses in macrophages (Butcher et al., 2018). The immune cells, non-immune cells at the maternal-fetal interface express TLRs, and the expression patterns are dependent of the stage of pregnancy in mice and humans (Koga and Mor, 2010). The maternal-fetal interface enhances tolerance to the allogenic fetus, and maintains innate immune responses against microorganisms, which are related to the TLRs in mice and humans (Abrahams and Mor, 2005). TLRs are involved in the immune regulation of the female reproductive tract, and the expressions of TLRs in different tissues are related to pregnancy outcome in mice and humans (Amirchaghmaghi et al., 2013). Expression of TLRs in the trophoblasts has immunological role at the maternal-fetal interface, and expressions of some components of TLRs are regulated by embryo- and/or pregnancy-related factors during early pregnancy in ewes (Kaya et al., 2017). There are changes in expression levels of the TLRs during PGF2α-induced luteolysis in CL, which is beneficial for the establishment of pregnancy in ewes (Atli et al., 2018). In addition, it has been reported that there are changes in expression of TLR2, TLR3, TLR4, and TLR5 in the ovine thymus during early pregnancy (Li et al., 2020). Therefore, we hypothesize that early pregnancy regulates expression of TLR signaling members in the maternal spleen. The objectives of this study were to detect the expression of TLR2, TLR3, TLR4, TLR5, TLR7, TLR9, myeloid differentiation primary-response protein 88 (MyD88), TNF receptor associated factor 6 (TRAF6) and IL-1-receptor-associated kinase 1 (IRAK1) in the maternal spleen from non-pregnant ewes and early pregnant ewes. The results will be useful for investigating the maternal immune tolerance during early pregnancy in mammals.

## Methods

### Experimental design and spleen collection

The study protocol was reviewed and approved by the Hebei University of Engineering Animal Care and Use Committee (AEEI-16015), and humane animal care and handling procedures were followed throughout the experiment. Small-tail Han ewes (approximately 18-month-old) with normal estrous cycles (average of 17 days) were selected and randomly divided into four groups. The ewes were housed at Handan Boyuan Animal Husbandry Co., Ltd. (Handan, China). The effects of early pregnancy on the ovine spleen are mainly due to P4 and IFNT as described previously (Bai et al., 2020). The ewes from days 13, 16 and 25 of pregnancy (DP13, DP16 and DP25) were bred with intact rams, after detection of sexual receptivity (day 0 of pregnancy or nonpregnancy) with vasectomized rams. The nonpregnant ewes were not mated with an intact ram. The spleens were collected from day 16 of nonpregnancy (DN16), DP13, DP16 and DP25 (n = 6 for each group) after the ewes were euthanatized. Pregnancy was detected through finding a conceptus in the uterus. The spleen samples were cut into small pieces, and immediately frozen in liquid nitrogen before real-time quantitative PCR (RT-qPCR) assay and western blot analysis. Some samples were fixed in fresh 4% (w/v) paraformaldehyde for following immunohistochemistry analysis.

### Gene expression analysis related with TLR signaling pathways

Total RNA was extracted from the splenic samples using TRIzol (Tiangen Biotech Co., Ltd., Beijing, China) following the manufacturer’s instructions. Quantity and quality of total RNA were verified using agarose gel (1%) electrophoresis and spectrometry (A260/A280, NanoDrop 2000). First-strand cDNA was synthesized by a FastQuant RT kit with DNase (Tiangen Biotech Co., Ltd.) according to the instruction. Primers (Table 1) for the target genes (*TLR2*, *TLR3*, *TLR4*, *TLR5*, *TLR7*, *TLR9*, *MYD88*, *TIRAP6* and *IRAK1*) and the reference gene (*GAPDH*) were designed and synthesized by Sangon Biotech Biotech Co., Ltd. (Shanghai, China). Thermal cycling conditions consisted of 40 cycles of 95 °C for 10 s, 60 to 62 °C (60 °C for *TLR2*, *TLR3*, *TLR4*, *TLR7* and *TRAF6*, or 62 °C for *TLR5*, *TLR9*, *IRAK1* and *MYD88*) for 20 s, and 72 °C for 25 s on a Bio-Rad CFX96 real-time PCR system (Bio-Rad Laboratories, Inc., CA, USA). Real-time qPCR was performed in triplicate using a SuperReal PreMix Plus kit (Tiangen Biotech Co., Ltd.) according to optimized PCR protocols and MIQE guidelines, and the reference gene was amplified in parallel with the target genes. The 2^-ΔΔCt^ analysis method (Schmittgen and Livak, 2008) was used to calculate relative expression levels that were normalized to the reference gene. The data from the ewes of DN16 was used as the normalization control.

**Table 1:** Primers used for RT-qPCR.

**Gene**	**Primer**	**Sequence**	**Size (bp)**	**Accession numbers**
*TLR2*	Forward	GGAGCGAGTGGTGCAAGTATGAG	200	NM_001048231.1
	Reverse	CCAGAATGCTTCCTGCTGAGTCTC		
*TLR3*	Forward	TCTCTGAACAATGCCAAGCTGAGC	123	NM_001135928.1
	Reverse	GGTCGTGTGGCTGATTGTGTCC		
*TLR4*	Forward	TGTGAAGGACATGCCAGTGCTTG	80	NM_001135930.1
	Reverse	TGACAACCGACACGCTGATGATC		
*TLR5*	Forward	CGTGTGGAGCAGCAGGAAGAC	116	NM_001135926.1
	Reverse	CGCCGTTGAGGTCAGCTAAGC		
*TLR7*	Forward	CCTGGAGGTATTCCTGCCAATGC	83	NM_001135059.1
	Reverse	CGGTGGAAGGAGGCTGGAGAG		
*TLR9*	Forward	CAAGGATGTCGTGGTGCTGGTG	183	NM_001011555.1
	Reverse	CGGTTATAGAAGTGGCGGTTGTCC		
*MYD88*	Forward	GCGAGGACGTGCTGATGGAAC	82	NM_001166183.1
	Reverse	GATGCCTCCTGCTGCTGCTTC		
*TRAF6*	Forward	AACTGAGGCATCTTGAGGAGCATC	82	XM_015101111.2
	Reverse	TTCTGGAAGAGACGCTGGCATTG		
*IRAK1*	Forward	GCTACTGTGCTCAGAGTGGCTTC	82	XM_027962961.1
	Reverse	TGGACGTGGAGGCGGTCTTC		
*GAPDH*	Forward	GGGTCATCATCTCTGCACCT	176	NM_001190390.1
	Reverse	GGTCATAAGTCCCTCCACGA		

### Protein expression analysis related with TLR signaling pathways

The splenic samples were homogenized in RIPA Lysis Buffer (Biosharp, BL504A), and total protein concentration was calculated using a BCA Protein Assay kit (Tiangen Biotech Co., Ltd.) with bovine serum albumin as the standard. The proteins (10 μg/lane) were separated by electrophoresis on 12% SDS-PAGE gels, and were transferred onto polyvinylidene fluoride membranes (Millipore, Bedford, MA, USA). The membranes were blocked with 5% fat-free milk at 4 °C overnight, and then incubated with a rabbit anti-TLR2 polyclonal antibody (Abcam, Cambridge, UK, ab191458, 1:1000), a mouse anti-TLR3 monoclonal antibody (Santa Cruz Biotechnology, Santa Cruz, CA, USA, sc-32232, 1:1000), a mouse anti-TLR4 monoclonal antibody (Santa Cruz Biotechnology, sc-293072, 1:1000), a mouse anti-TLR5 monoclonal antibody (Santa Cruz Biotechnology, sc-517439, 1:1000), a rabbit anti-TLR7 polyclonal antibody (Abcam, ab113524, 1:1000), a mouse anti-TLR9 monoclonal antibody (Santa Cruz Biotechnology, sc-52966, 1:1000), a mouse anti-MyD88 monoclonal antibody (Santa Cruz Biotechnology, sc-136970, 1:1000), a mouse anti-TRAF6 monoclonal antibody (Santa Cruz Biotechnology, sc-8409, 1:1000) and a rabbit anti-IRAK1 polyclonal antibody (Abcam, ab137327, 1:1000), respectively. A horseradish peroxidase (HRP) conjugated goat anti-mouse (Biosharp, BL001A) or anti-rabbit IgG (Biosharp, BL003) was used at a 1:10,000 dilution. Immunoreactive proteins were detected using a pro-light HRP chemiluminescence kit (Tiangen Biotech Co., Ltd.). An anti-GAPDH antibody (Santa Cruz Biotechnology, sc-47724, 1:1000) was used to normalize the relative expression levels of target proteins. The band intensities were quantified by the Quantity One software (Bio-Rad Laboratories).

### Immunolocalization for MyD88

The fixed splenic tissues were dehydrated in ethanol, and embedded in paraffin. The paraffin-embedded samples were cut to 5 μm-thick sections. Several sections were stained by hematoxylin and eosin (HE) after deparaffinization and rehydration. Other sections were treated with boiling citrate for antigen retrieval. Endogenous peroxidase activity was blocked with 3% H_2_O_2_, and nonspecific binding was reduced with 5% normal goat serum. Immunohistochemical localization of MyD88 protein in the spleen was performed using the mouse anti-MyD88 monoclonal antibody (Santa Cruz Biotechnology, sc-136970, 1:200). Negative controls were treated with purified non-immune mouse immunoglobulin G instead of the anti-MyD88 antibody at the same concentration. A DAB kit (Tiangen Biotech Co., Ltd.) was used to detect the MyD88 protein, and the nuclei were stained with hematoxylin. Photomicrographs were taken using a light microscope (Nikon Eclipse E800, Japan) with a digital camera (AxioCam ERc 5s). Finally, the photomicrographs were analyzed by two investigators in a blinded fashion (Kandil et al., 2007).

### Statistical analysis for the data

The data for relative expression levels of mRNA and proteins of TLR2, TLR3, TLR4, TLR5, TLR7, TLR9, IRAK1, TRAF6 and MYD88 were analyzed using the mixed procedure of the Statistical Analysis System (SAS Institute, Cary, NC). ANOVA followed by Tukey’s post hoc test were used to compare the relative expression levels of the different groups, and the data were normally distributed. Data are presented as least squares means. A *P* < 0.05 value was considered statistically significant.

## Results

### Early pregnancy changes expressions of *TLR2*, *TLR3*, *TLR4*, *TLR5*, *TLR7*, *TLR9*, *IRAK1*, *TRAF6* and *MYD88* mRNA in the spleens

**Figure 1:**
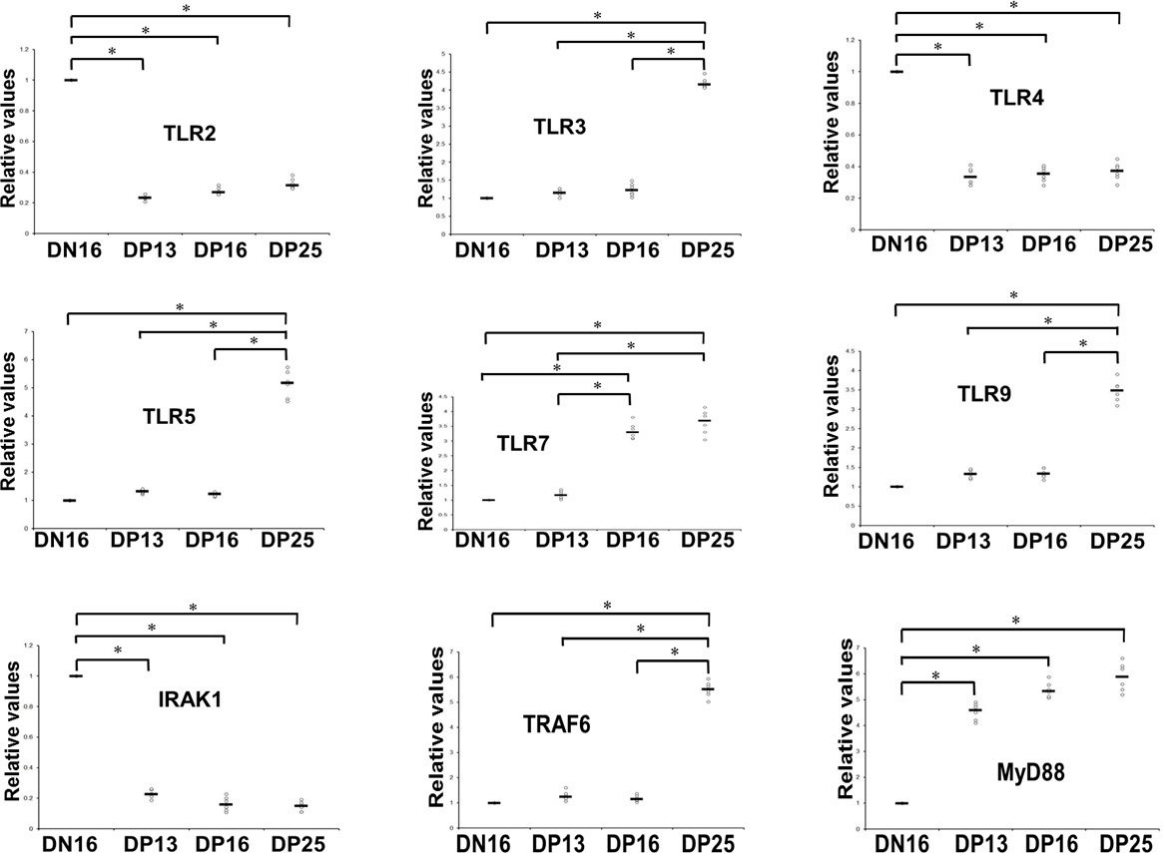
Relative expression values of *TLR2, TLR3, TLR4, TLR5, TLR7, TLR9, IRAK1, TRAF6* and *MYD88* mRNA in ovine spleens measured by quantitative real-time PCR (n = 6 for each group). Note: DN16 = day 16 of the estrous cycle; DP13 = day 13 of pregnancy; DP16 = day 16 of pregnancy; DP25 = day 25 of pregnancy. Significant differences (*P* < 0.05) are indicated by *.

The data of RT-qPCR showed that relative expression levels of *TLR2*, *TLR4* and *IRAK1* mRNA were downregulated, but *MYD88* mRNA level was increased during early pregnancy (*P* < 0.05; Figure 1). Furthermore, there was an upregulation of relative expression level of *TLR7* mRNA in DP16 and DP25. The levels of *TLR3*, *TLR5, TLR9* and *TRAF6* mRNA were increased in DP25 (*P* < 0.05; Figure 1).

### Early pregnancy affects expressions of TLR2, TLR3, TLR4, TLR5, TLR7, TLR9, IRAK1, TRAF6 and MyD88 proteins in the spleens

**Figure 2:**
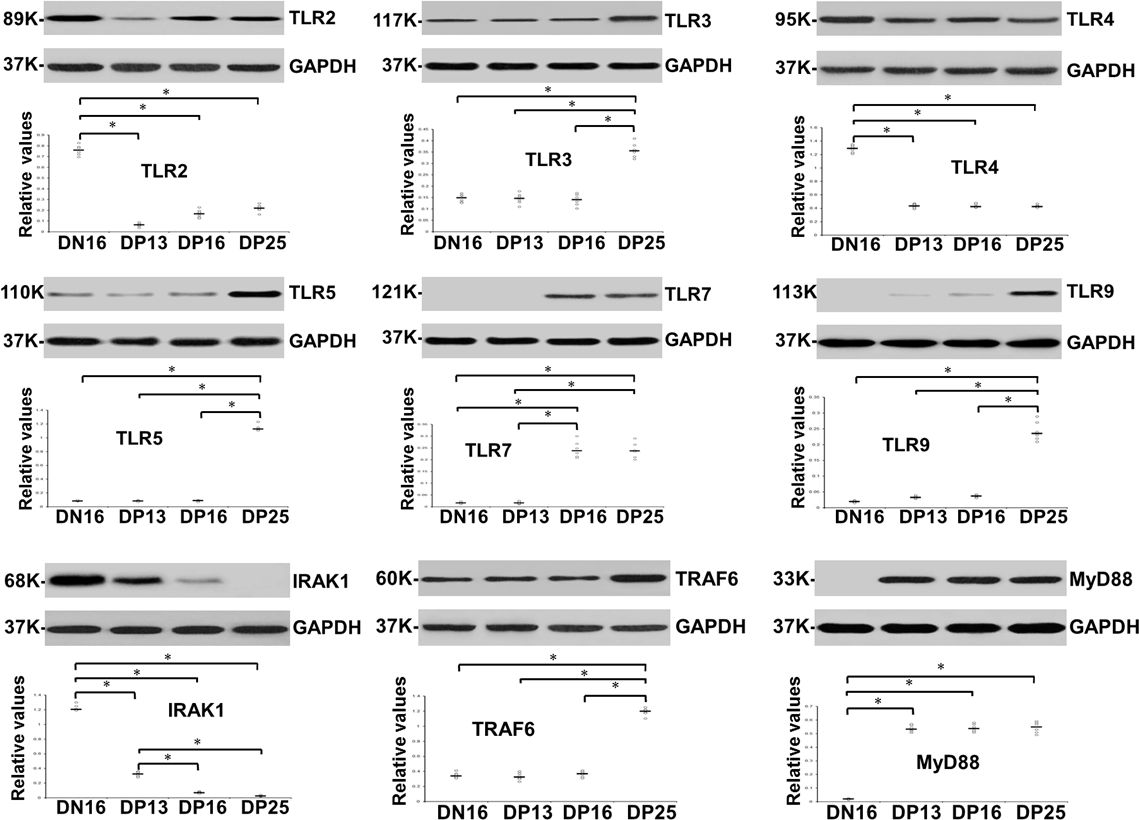
Expression of TLR2, TLR3, TLR4, TLR5, TLR7, TLR9, IRAK1, TRAF6 and MyD88 proteins in ovine spleens analyzed by western blot (n = 6 for each group). Note: DN16 = day 16 of the estrous cycle; DP13 = day 13 of pregnancy; DP16 = day 16 of pregnancy; DP25 = day 25 of pregnancy. Significant differences (*P* < 0.05) are indicated by *.

Western blot analysis revealed (Figure 2) that early pregnancy induced downregulation of TLR2, TLR4 and IRAK1 proteins in the maternal spleens (*P* < 0.05; Figure 2), and there was no expression of IRAK1 protein in DP25. However, MyD88 protein was expressed during early pregnancy, and undetectable in DN16. Furthermore, TLR7 protein was enhanced in DP16 and DP25, but there was no expression in DN16 and DP13. In addition, TLR3, TLR5, TLR9 and TRAF6 proteins were upregulated in DP25 (*P* < 0.05), and TLR9 protein was undetectable in DN16.

### Early pregnancy changes location of MyD88 protein in the spleens

**Figure 3:**
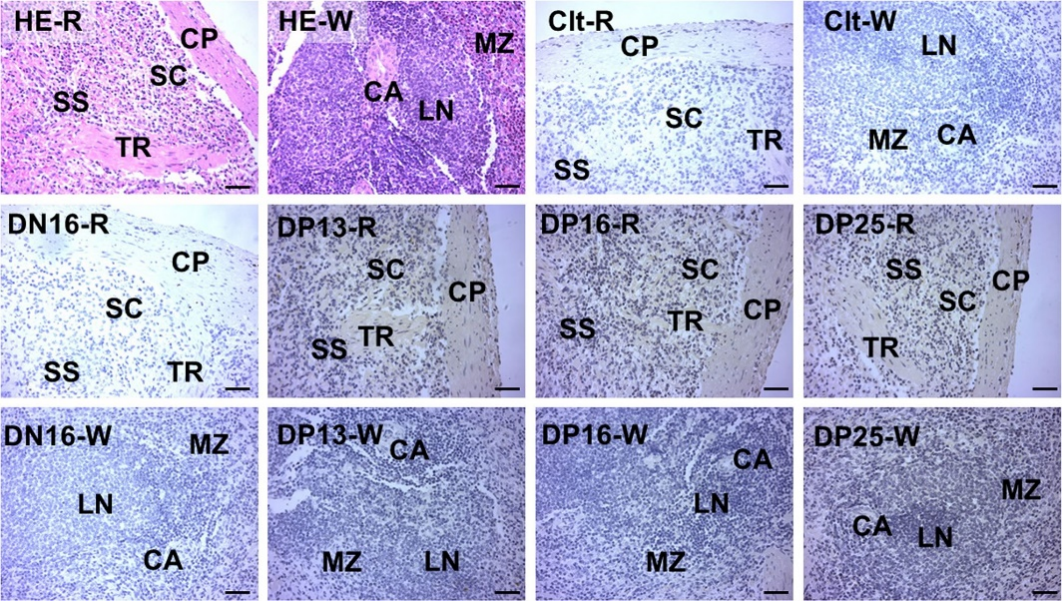
Representative immunohistochemical localization of MyD88 protein in the ovine spleens. The spleen is divided into red pulp (R) and white pulp (W), and surrounded by a thickened capsule. Capsule (CP) with several trabeculae (TR) projects into the substance of the spleen. Note: HE = stained by hematoxylin and eosin; Clt = negative control; SS = splenic sinuses; SC = splenic cords; MZ = marginal zone; LN = lymphoid nodule; CA = central artery; DN16 = day 16 of the estrous cycle; DP13 = day 13 of pregnancy; DP16 = day 16 of pregnancy; DP25 = day 25 of pregnancy. Bar = 50 µm.

It was showed in the Figure 3 that there is no stain in the negative control and the spleens from DN16. However, the staining intensities for MyD88 protein were stronger in the spleens from DP13, DP16 and DP25 comparing with that from DN16. In addition, the MyD88 protein was located in the capsule, trabeculae and splenic cords (Figure 3).

## Discussion

It is reported for the first time that early pregnancy regulates TLR-mediated signaling in the maternal spleen. In this study, early pregnancy inhibited the expression of TLR2 in the ovine spleen. TLR2 affects T cell polarization and differentiation, and plays a crucial role in development of experimental autoimmune disease (Borrello et al., 2011). Pre-eclampsia induces upregulation of neutrophil *TLR2* mRNA and protein comparing with normal pregnancy in women (Xie et al., 2010). Gene expression level of *TLR2* in neutrophil is enhanced from the women with preterm than that from the normal control group, which indicates that TLR2 is related with the activation of inflammatory response at the maternal-fetal interface (Prearo Moço et al., 2018). The P4 concentration is low on days 15 and 16 during ovine estrous cycle (Mcnatty et al., 1973), and CL secretes P4 during early pregnancy. It is suggested that downregulation of TLR2 in the maternal spleen may be due to high level of P4 during early pregnancy in sheep.

Our results revealed that there was an upregulation of *TLR3* mRNA and protein at day 25 of pregnancy in the ovine spleen. TLR3 plays key roles in maintaining tolerance or inducing immune responses through modulating T-cell responses (Salio and Cerundolo, 2005). Indoleamine 2,3-dioxygenase (IDO) participates in suppressing fetal rejection in mice. TLR3 induces IDO expression in human first-trimester trophoblasts to participate in maintenance of fetomaternal tolerance (Wang et al., 2011). Pregnancy-associated glycoproteins (PAGs) are produced by the ovine placenta from day 20 of pregnancy (Karen et al., 2003). It is supposed that upregulation of TLR3 in maternal spleen on day 25 of pregnancy may be related to PAGs secretion in sheep.

Our data manifested that early pregnancy induced downregulation of *TLR4* mRNA and protein in the maternal spleen. TLR4 activation in the placental tissue is related to infant morbidity and mortality, and inhibition of functional TLR4 results in downregulation of the pro-inflammatory responses, which enhances pregnancy outcome in mice (Barboza et al., 2017). There is an upregulation of *TLR4* gene in the neutrophils from the women with preterm comparing to the normal control group, suggesting that upregulation of TLR4 in maternal neutrophils participates in spontaneous preterm labor in humans (Prearo Moço et al., 2018). There is a low concentration of P4 on days 15 and 16 of nonpregnancy (Mcnatty et al., 1973), and CL secretes P4 during early pregnancy. It is suggested that downregulation of TLR4 during early pregnancy may be related to high level of serum P4 in sheep.

It was found in this study that early pregnancy stimulated expression of *TLR5* mRNA and protein at day 25 of pregnancy in the spleen. TLR5 is necessary for flagellin-specific adaptive immunity through improving the presentation of peptides to flagellin-specific CD4^+^ T cells (Letran et al., 2011). Expression of *TLR5* mRNA in the endometrium is upregulated with increasing doses of P4 at mid- to late pregnancy in pigs (Yoo et al., 2019). It is known that there are low level of estradiol and high level of P4 during early pregnancy, and PAGs levels are increased from day 20 of pregnancy in ewes (Karen et al., 2003). Therefore, it is suggested that the upregulation of TLR5 in the maternal spleen at day 25 of pregnancy may be related to low level of estradiol, and high levels of P4 and PAGs in the plasma of ewes.

Our data indicated that there was an upregulation of *TLR7* mRNA and protein at days 16 and 25 of pregnancy in the spleen. TLR7 participates in the maintenance of autoimmunity in the pathogenesis of experimental autoimmune encephalomyelitis in mice (Lalive et al., 2014). TLR7 is expressed in uterine epithelia and stroma, and conceptuses, which are involved in conceptus development and establishment of pregnancy during peri-implantation period in sheep (Ruiz-González et al., 2015). TLR7 expression is upregulated during early pregnancy, and TLR7 protein is particularly localized in endothelial cells of the CL in sheep (Atli et al., 2018). IFNT is detectable between days 14 and 21 of pregnancy (Godkin et al., 1982), and PAGs are secreted by the placenta from day 20 of pregnancy in sheep (Karen et al., 2003). It is supposed that the upregulation of TLR7 in maternal spleen at days 16 and 25 of pregnancy may be related to IFNT and PAGs in sheep.

Our results demonstrated that early pregnancy induced expression of *TLR9* mRNA and protein in the maternal spleen at day 25 of pregnancy. TLR9 signaling is involved in immune activation and autoantibody production (Vollmer, 2006). It has been reported that TLR9 is downregulated in splenic B cells from abortion-prone mice comparing to that from normal animals at day 14 post-coitum (Lorek et al., 2019). PAGs are produced by the ovine placenta from day 20 of pregnancy (Karen et al., 2003). It is suggested that upregulation of TLR9 in maternal spleen at day 25 of pregnancy may be related to PAGs in sheep.

Our results showed that there was a downregulation of *IRAK1* mRNA and protein in the maternal spleen during early pregnancy. Expression of *IRAK1* mRNA is increased in the deciduae of patients with unexplained recurrent spontaneous abortion compared with that in healthy women, which is the cause of the immune intolerance of fetal-placental unit (Zhao et al., 2018). Systemic inflammation induces an increased expression level of IRAK1 in maternal spleen of mice (Lyttle et al., 2009). Sodium fluoride attenuates innate immunity through downregulation of *IRAK1* mRNA and protein in the mouse spleen (Kuang et al., 2019). It is indicated that downregulation of IRAK1 in the maternal spleen may be related to the decrease in innate immunity, which is necessary for the maternal immune tolerance in ewes.

In this study, there was an upregulation of *TRAF6* mRNA and protein in the maternal spleen at day 25 of pregnancy. TRAF6 is involved in regulating self-tolerance and autoimmunity in medullary thymic epithelial cells (Akiyama et al., 2008). TRAF6 participates in protein-protein interactions, and plays key roles in proper activation of the immune system and maintaining immune tolerance (Walsh et al., 2015). TLR3, TLR5 and TLR9 participate in TLR signaling pathways through activation of TRAF6 (Kawai and Akira, 2010). Therefore, the upregulation of TRAF6 may be caused by increased expressions of TLR3, TLR5 and TLR9 during early pregnancy, which is beneficial for maintaining immune tolerance.

Our data proved that expression of *MYD88* mRNA and protein was increased in the ovine maternal spleen during early pregnancy. Expression of *MYD88* mRNA is enhanced in the endometrium during pregnancy comparing to that during the estrous cycle in pigs (Yoo et al., 2019). MyD88 participates in numerous important pathways in innate immunity, and is necessary for the generation of an inflammatory response, and preventing excessive inflammation in the lung (Cervantes, 2017). It is suggested that upregulation of MyD88 in the maternal spleen may be co-caused by P4, IFNT and PAGs, which are favorable for pregnancy establishment in sheep.

As a secondary lymphatic organ, the spleen is divided into red and white pulps, and involved in immune surveillance of the blood, lymphocyte recirculation and the final steps of B-cell maturation (Steiniger, 2015). Our immunohistochemistry results revealed that MyD88 protein was mainly located in capsule, trabeculae and splenic cords, and the staining intensities for MyD88 were stronger in the splenic tissues from the pregnant ewes. Red pulp includes an open circulation, and closely contacts with circulating blood cells (Nagelkerke et al., 2018). The spleen is responsible for initiating immune reactions to blood-borne antigens, and participates in systemic immune responses (Cesta, 2006). Therefore, the upregulation of MyD88 in the capsule, trabeculae and splenic cords may be helpful for regulating maternal systemic immune responses during early pregnancy in sheep.

## Conclusion

In summary, early pregnancy inhibited expression of TLR2, TLR4 and IRAK1, but enhanced expression of TLR3, TLR5, TLR7, TLR9, TRAF6 and MyD88 in the ovine maternal spleen. Furthermore, the MyD88 protein was located in capsule, trabeculae and splenic cords. This paper reports for the first time that there are changes in TLR signaling pathways in maternal spleen, which may be helpful for understanding the maternal immune tolerance during early pregnancy. Future studies may pay more attention for the pregnancy immune tolerance.
